# Differential activation of JNK1 isoforms by TRAIL receptors modulate apoptosis of colon cancer cell lines

**DOI:** 10.1038/sj.bjc.6605021

**Published:** 2009-04-07

**Authors:** D Mahalingam, M Keane, G Pirianov, H Mehmet, A Samali, E Szegezdi

**Affiliations:** 1 Cell Stress and Apoptosis Research Group, Department of Biochemistry and National Centre of Biomedical Engineering Science, National University of Ireland, University Road, Galway, Ireland; 2 Department of Medicine, University College Hospital, Galway, Ireland; 3 Institute of Reproductive and Developmental Biology, Faculty of Medicine, Imperial College London, Hammersmith Hospital Campus, Du Cane Road, London W12 0NN, UK

**Keywords:** TRAIL, c-Jun, JNK1*α*1, colon carcinoma, apoptosis, DR4, DR5

## Abstract

Tumour necrosis factor-related apoptosis-inducing ligand (TRAIL) induces apoptosis on binding to its receptors, death receptor 4 and 5 (DR4, DR5). TRAIL can also activate c-Jun N-terminal kinase (JNK) through the adaptor molecules, TNF receptor-associated factor 2 (TRAF2) and receptor-interacting protein (RIP). The role of JNK in TRAIL-induced tumour cell apoptosis is unclear. In this study, we demonstrate that JNK is activated by TRAIL in colon cancer cells. Inhibition of JNK with L-JNKI reduced rhTRAIL-induced cell death but enhanced cell death induced by selective activation of DR4 or DR5. This difference was unrelated to receptor internalisation or differential activation of c-Jun, but activation of different JNK isoforms. Our data demonstrate that JNK1, but not JNK2 is activated by rhTRAIL in the examined colon cancer cell lines. Although rhTRAIL activated both the long and short isoforms of JNK1, selective activation of DR4 or DR5 led to predominant activation of the short JNK1 isoforms (JNK1*α*1 and/or JNK1*β*1). Knockdown of JNK1*α*1 by shRNA enhanced apoptosis induced by TRAIL, agonistic DR4 or DR5 antibodies. On the other hand, knockdown of the long JNK1 isoforms (JNK1*α*2 and JNK1*β*2) had the opposite effect; it reduced TRAIL-induced cell death. These data indicate that the short JNK1 isoforms transmit an antiapoptotic signal, whereas the long isoforms (JNK1*α*2 or JNK1*β*2) act in a proapoptotic manner.

Tumour necrosis factor-related apoptosis-inducing ligand (TRAIL) is a member of the TNF ligand superfamily (Ashkenazi and Dixit, 1998). TRAIL induces apoptosis on binding to its transmembrane, death domain-containing receptors, TRAIL receptors 1 (death receptor 4; DR4) and 2 (death receptor 5; DR5). Three other TRAIL-binding receptors exist, but they are unable to transmit an apoptotic signal and thus considered to be ‘decoy receptors’. Decoy receptor 1 (DcR1) lacks the transmembrane and intracellular domains and is anchored to the plasma membrane via a glycosylphosphatidylinositol-tail. Decoy receptor 2 (DcR2) possesses a truncated, non-functional death domain, whereas the third decoy receptor, osteoprotegerin is a secreted, soluble receptor (Ashkenazi and Dixit, 1998).

Binding of homotrimeric TRAIL to DR4 and DR5 induces receptor trimerisation and activation leading to recruitment of adaptor proteins and formation of the death-inducing signalling complex (DISC). Procaspase-8 and/or -10 are recruited to the DISC, leading to their oligomerisation and activation. Active caspase-8/-10 can activate the executioner caspases (procaspase-3, -6 and -7) and/or initiate the mitochondrial apoptotic pathway by cleaving the BH3-only protein Bid.

Generally, caspase activation is the main outcome following activation of DR4/5 by TRAIL. However, TRAIL, via adaptor molecules such as TNF receptor-associated factor 2 (TRAF2), receptor-interacting protein (RIP), and the mitogen-activated protein kinase kinases (MKK)-4 and -7, can also activate the c-Jun N-terminal kinase (JNK) pathway (Hu *et al*, 1999; Lin *et al*, 2000). JNK activation is also regulated by scaffold proteins, JNK-interacting protein (JIP) and JNK stress-activated protein kinase-associated protein 1 (JSAP1) (Whitmarsh *et al*, 1998; Ito *et al*, 1999). Depending on the cell type and the stimulus, JNK can activate a number of diverse downstream targets including members of the activator protein-1 (AP-1) family, c-Jun, JunD, activating transcriptional factor 2 (ATF2), Bcl-2 proteins, c-Myc and p53 (Bode and Dong, 2007; Lin *et al*, 2007). Whether JNK induces or suppresses apoptosis is largely dependent on the molecules it activates. For example, JNK can both phosphorylate antiapoptotic Bcl-2 proteins to promote apoptosis or phosphorylate proapoptotic Bcl-2 proteins (e.g. BAD) to inhibit apoptosis (Maundrell *et al*, 1997; Yu *et al*, 2004).

JNK proteins are encoded by three genes, *jnk1*, *jnk2* and *jnk3*. *Jnk1* and *jnk2* encode ubiquitously expressed JNK proteins whereas the *jnk3* protein product is primarily found in the brain, heart and to a lesser extent in the testis (Bode and Dong, 2007). Alternative splicing of the *jnk* transcripts results in 10 different JNK isoforms each of which may target different transcription factors (Gupta *et al*, 1996). JNK1 and -2 have four isoforms each (*α*1, *α*2, *β*1 and *β*2). JNK1*α*1, JNK1*β*1, JNK2*α*1 and JNK2*β*1 isoforms all have a molecular weight of 46 kDa, whereas JNK1*α*2, JNK1*β*2, JNK2*α*2 and JNK2*β*2 isoforms are larger due to an extended C-terminus and their molecular weight is 54 kDa. JNK3 has two isoforms, JNK3*α*1 (46 kDa) and JNK3*α*2 (54 kDa). The *α*- and *β*-isoforms correspond to two alternative stretches of sequences in the kinase subdomains IX and X (Gupta *et al*, 1996).

The mechanism and role of JNK activation in TRAIL-induced tumour cell apoptosis has not been fully elucidated. Some reports suggest that JNK is not activated by TRAIL in colon cancers regardless of their sensitivity to TRAIL (Zhang *et al*, 2004), whereas others suggest that JNK activation augments TRAIL-induced apoptosis in a number of other tumours (Herr *et al*, 1999; Li *et al*, 2006; Mikami *et al*, 2006). The reason for this discrepancy is currently not known. It was therefore of interest to investigate which JNK isoforms are activated by which TRAIL receptor and how the different JNK isoforms contribute to TRAIL-induced colon cancer cell death.

## Materials and methods

### Cell culture and treatments

Colo205 cells were obtained from American Tissue Culture Collection (ATCC, Manassas, VA, USA). HCT15 and HCA7 cells were a kind gift from Professor L Egan (University College Hospital, Galway). Colo205 and HCT15 cells were maintained in RPMI-1640 medium and HCA7 in DMEM medium, both media supplemented with 10% fetal bovine serum (FBS), 2 mM glutamine, 50 U ml^−1^ penicillin and 50 mg ml^−1^ streptomycin at 37°C, 5% CO_2_ in a humidified incubator. Cells were treated with rhTRAIL (non-tagged, fragment of amino acids 114–281; Triskel Therapeutics, Groningen, The Netherlands), agonistic anti-DR4 or anti-DR5 antibodies (Novartis Pharmaceuticals, Basel, Switzerland). To inhibit JNK activation, L-JNKI (Calbiochem, San Diego, CA, USA), a cell-permeable, 21-amino-acid peptide inhibitor of activated JNK was added 30 min before treatment with TRAIL or agonistic antibodies. UV treatment was done at 25 J m^−2^ for 3 min as a positive control. All reagents were from Sigma-Aldrich (St Louis, MO, USA) unless otherwise stated.

### Cell viability assay

Cell viability was monitored by 2-(4, 5-dimethyltriazol-2-yl)-2, 5-diphenyl tetrazolium bromide (MTT) assay as described before (Szegezdi *et al*, 2006).

### Cell death assay

Cell death was monitored by labelling phosphatidyl serine on the surface of apoptotic cells with Annexin-V-FITC. Following treatment, cells were collected by gentle trypsinisation and incubated for 10 min at 37°C to allow membrane recovery after trypsinisation, pelleted by centrifugation at 350 × **g** and incubated with Annexin-V-FITC in calcium buffer (10 mM HEPES/NaOH, pH 7.4, 140 mM NaCl and 2.5 mM CaCl_2_) for 15 min on ice in the dark. A wash step in calcium buffer was carried out before acquisition on a FacsCalibur flow cytometer (Becton Dickinson, Oxford, England).

### Protein lysate preparation and western blot analysis

Cells were lysed in a buffer containing 1% Triton X-100, 100 mM Tris/HCl pH 8.0, 200 mM sodium chloride (NaCl), 5 mM EDTA, 10% glycerol, 1 mM dithiothreitol (DTT), 1 mM phenylmethylsulphonyl fluoride (PMSF), 5 *μ*g ml^−1^ aprotinin, 2.5 *μ*g ml^−1^ leupeptin, 1 mM sodium orthovanadate (Na_2_VO_3_) and 1 mM sodium fluoride (NaF). Cellular proteins (30 *μ*g) were separated by electrophoresis on 10% SDS polyacrylamide gel and transferred onto nitrocellulose membranes. Blots were incubated with rabbit monoclonal antibodies to total JNK, phospho (p)-JNK (1 : 1000; Cell Signaling Technology, Beverly, MA, USA), JNK1 (1 : 1000; BD Pharmigen, San Diego, CA, USA) or mouse monoclonal antibodies to p-JNK (1 : 500), Caspase-8 (1 : 1000; Cell Signaling Technology) and JNK2 (1 : 1000; Santa Cruz Technologies, Santa Cruz, CA, USA). For detection, the appropriate horseradish peroxidase-conjugated goat secondary antibodies were used. Protein bands were visualised with SuperSignal West Pico Chemiluminescent Substrate (Pierce, Rockford, IL, USA) on X-ray film (Agfa, Mortsel, Belgium).

### *In-vitro* kinase assay (GST p-c-Jun)

JNK activity was measured using a specific kit (Cell Signaling Technology) following the manufacturer's instructions and using GST fusion peptide as the specific substrate for JNK. In brief, cell lysates (100 *μ*g protein) were incubated overnight at 4°C with GST-c-Jun fusion protein beads. After washing, the beads were resuspended in kinase buffer containing ATP and kinase reaction was allow to proceed for 30 min at 30°C. Reactions were stopped by the addition of polyacrylamide gel electrophoresis (PAGE) sample loading buffer. Proteins were separated by electrophoresis on a 10% PAGE gel, transferred on PVDF membrane and incubated with phospho-c-Jun (Ser63) antibody. Finally, blots were subjected to enhanced chemiluminescence and kinase activity determined by densitometric analysis.

### Cell surface expression of TRAIL receptors

Cells were washed twice in PBS containing 1% BSA and then incubated with monoclonal antibodies to DR4 or DR5 (Alexis, Lausen, Switzerland) for 40 min. After two wash steps with PBS/BSA, anti-mouse IgG-FITC (Sigma) secondary antibody was added for 30 min. All incubations were carried out on ice. Negative controls contained isotype control antibody. Cells were analysed by FacsCalibur flow cytometer (Becton Dickinson).

### Measurement of receptor internalisation by flow cytometric analysis

To measure cellular uptake of receptor bound TRAIL and agonistic DR4/5 antibody, 2 × 10^5^ Colo205 cells were incubated at 4 or 37°C in the presence of 50 ng ml^−1^ FITC-conjugated TRAIL or agonistic DR4/5 antibody cross-linked with FITC-labelled anti-mouse antibody for 30 min. Samples were rapidly chilled on ice to inhibit endocytosis and cells were collected by a brief centrifugation at 4°C. After washing twice in prechilled wash buffer (20 mM Hepes, pH 7.4, 150 mM NaCl, 5 mM KCl, 1 mM CaCl_2_, 1 mM MgCl_2_), cell surface-bound ligand/antibody was removed by resuspension in prechilled acid wash solution (0.2 M NaCl, 0.2 M acetic acid) for 5 min on ice. Cells were subsequently washed three times in wash buffer and resuspended in cold PBS containing 2% (w/v) FBS before immediate quantification of ligand internalisation using a FacsCalibur flow cytometer (Becton Dickinson).

### Immunoprecipitation of JNK

Cells were lysed in phosphate lysis buffer (PLB) containing 20 mM sodium phosphate, 137 mM NaCl, 25 mM sodium-*β*-glycerol phosphate, 2 mM sodium pyrophosphate, 2 mM EDTA, 1% Triton X-100, 10% glycerol, 1 mM DTT, 1 mM PMSF, 5 *μ*g ml^−1^ aprotinin, 2.5 *μ*g ml^−1^ leupeptin, 1 mM Na_2_VO_3_ and 1 mM NaF with sonication. To cross-link mouse monoclonal JNK1 (BD Pharmigen) and p-JNK (Cell Signaling Technology) antibodies to protein G-sepharose beads (Sigma), 2 *μ*g antibody was incubated with 30 *μ*l of beads for 1 h at 4°C, washed twice with PLB, resuspended in 0.2 M sodium borate pH 9.0 containing 20 mM dimethylpimelidate (DMP) and incubated for 45 min at room temperature. The reaction was stopped by washing the beads once with 0.2 M ethanolamine/HCl pH 8.0 and incubating at room temperature for 2 h. The beads were washed twice in 200 mM glycine and 200 mM NaCl pH 2.5 followed by a wash in 500 mM Tris/HCl pH 8.0. Protein (300 *μ*g) was incubated for 2 h at 4°C with cross-linked JNK1 or p-JNK (all isoforms) antibodies bound to protein G-Sepharose. After two rounds of washes with PLB, the beads were resuspended in 30 *μ*l of 1X Laemmli buffer. The supernatant was loaded on a 10% SDS–PAGE acrylamide gel and transferred to nitrocellulose membranes. Membranes containing the JNK1 immunoprecipitates were incubated with mouse monoclonal JNK1 (BD Pharmigen) and rabbit monoclonal p-JNK (Cell Signaling Technology) antibodies to identify which JNK1 isoforms were activated. Membranes of the p-JNK immunoprecipitates were probed with JNK2 (Santa Cruz) and rabbit monoclonal total p-JNK (Cell Signaling Technology) antibodies to identify the JNK2 isoforms activated. Detection and visualisation was carried out as described above.

### Transient transfections and plasmids

Cells (2 × 10^6^) were pelleted and resuspended in transfection solution V (Amaxa, Walkersville, MD, USA) containing 2.5 *μ*g of mammalian JNK1*α*1 siRNA and the negative control expression plasmid, pKD-JNK1*α*1/SAPK1c-v1 (Millipore, Billercia, MA, USA; Catalogue no. 62-270, target sequence undisclosed) and pKD-NegCon-v1 (Catalogue no. 62-002; Millipore), or siRNA against JNK1*α*2/*β*2 and GFP as negative control by nucleofection using program T13 as per manufacturer's protocol (Amaxa). GFP plasmid (2.5 *μ*g) was also transfected to analyse transfection efficiencies. Twenty-four hours after transfection, cells were resuspended in RPMI-1640 and seeded for Annexin V assays, RNA isolation or protein sampling. Sequence of JNK1*α*2/*β*2 siRNA1: sense–UUAGGUGCAGCAGUGAUCAtt; JNK1*α*2/*β*2 siRNA2: sense–UAGGUGCAGCAGUGAUCAAtt; GFP sense–GGCUACGUCCAGGAGCGCACCtt.

### RNA isolation, cDNA synthesis, RT–PCR

Total RNA was isolated from cells using the GenElute Mammalian Total RNA Extraction kit (Sigma). Reverse transcription was carried out with 2 *μ*g total RNA and oligo(dT) (Invitrogen, Paisley, Scotland) using 20 U/25 *μ*l reaction avian myeloblastosis virus reverse transcriptase. cDNAs for genes of interest were amplified during 32 cycles of 30 s denaturation at 94°C, 30 s annealing at 56°C, and 60 s extension at 72°C, with the following primers: JNK1*α*1 reverse – TCA CTG CTG CAC CTG TGC TAA AGG, forward – TGC CAC AAA ATC CTC TTT CCA GGA; JNK1 reverse – TCT TGG TTC TCT CCT CCA AGT C, forward – GTC AGG CAA GGG ATT TGT TAT; JNK1*β*1 reverse – ACT GCT GCA CCT GTG CTA AAG GAG, forward – AGG TGG TGT TTT GTT CCC AGG TAC, GAPDH was used as a loading control; GAPDH reverse – TCC ACC ACC CTG TTG CTG; forward – ACC ACA GTC CAT GCC ATC. The binding sites of the isoform specific primers in relation to the different JNK splice variants are depicted in Supplementary Figure 2.

### Statistical analysis

Differences in Annexin V staining between the treatment groups were analysed used a non-paired Student's *t*-test, with a significance of *P*<0.05. Error bars are shown as standard error of mean (s.e.m.). All statistical analysis was carried on Graphpad Prism 4 (GraphPad Software Inc., La Jolla, CA, USA).

## Results

### Colo205, HCT15 and HCA7 colon cancer cells are sensitive to TRAIL

**Figure 1:**
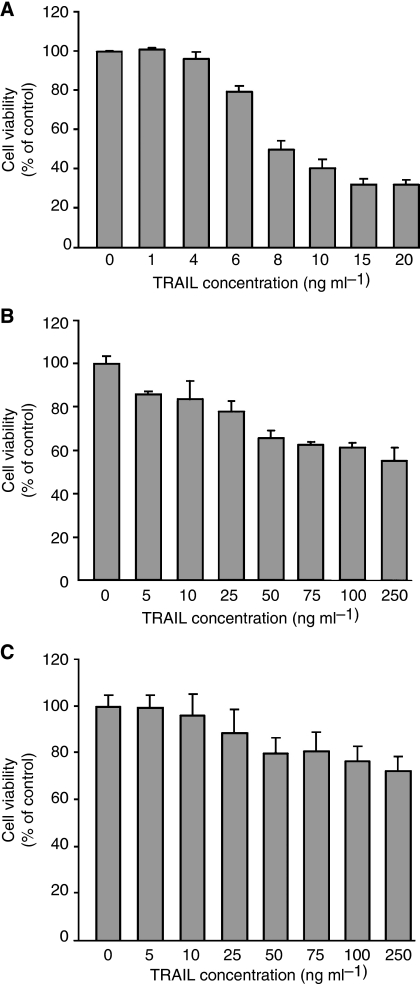
Colo205, HCT15 and HCA7 colon cancer cells are sensitive to TRAIL. (**A**) Colo205 cell viability after treatment with increasing concentration of rhTRAIL (0–20 ng ml^−1^) for 24 h measured by MTT assay. (**B**) HCT15 and (**C**) HCA7 cell viability after treatment with increasing concentration of rhTRAIL (0–250 ng ml^−1^) for 24 h measured by MTT assay. The graphs show average viability expressed as percentage of control±s.d. of three independent experiments.

**Figure 2:**
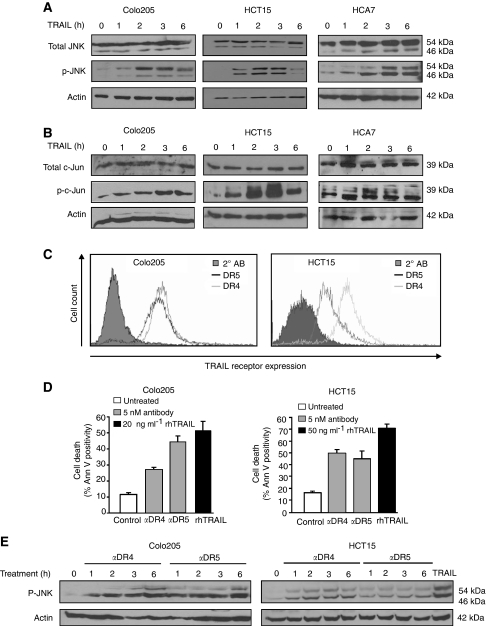
TRAIL can activate the JNK pathway via both DR4 and DR5 in colon cancer cell lines. (**A**) Western blot analysis of total JNK and p-JNK in Colo205, HCT15 and HCA7 cell lysates following treatment with 20 ng ml^−1^ rhTRAIL for Colo205 cells and 50 ng ml^−1^ for HCT15 and HCA7 for the times indicated. Total JNK levels and actin were also detected as loading controls. (**B**) Western blot analysis of total c-Jun and p-c-Jun levels in Colo205, HCT15 and HCA7 cell lysates following rhTRAIL treatment as above. (**C**) Cell surface expression of DR4 and DR5 in Colo205 and HCT15 cells measured by immunostaining followed by flow cytometry. Each histogram shows an overlay of a negative control (2° AB), DR4 and DR5 receptors. (**D**) Apoptosis-inducing potential of DR4 and DR5 in Colo205 and HCT15 cells. Cells were treated with cross-linked agonistic DR4 or DR5 antibodies (5 nM for Colo205 and 10 nM for HCT15) or rhTRAIL (20 and 50 ng ml^−1^ for Colo205 and HCT15) for 3 h in Colo205 cells and for 5 h in HCT15 cells. Apoptosis induction was measured with Annexin V. The graphs show averaged percentage of apoptotic cells±s.e.m. of three independent experiments. (**E**) Western blot analysis showing activation of JNK via DR4 and DR5 in Colo205 and HCT15 cells. Cells were treated with agonistic DR4 and DR5 antibodies (5 nM for Colo205 cells and 10 nM for HCT15 cells) for the times indicated. JNK phosphorylation was detected in whole cell lysates. The images shown are representatives of three independent experiments. Actin was detected as a loading control.

To examine the sensitivity of colon cancer cells to TRAIL, Colo205, HCT15 and HCA7 cells were treated with increasing concentrations of rhTRAIL for 24 h and cell viability assessed by MTT assay (Figure 1). All three cell lines express both DR4 and DR5 on their surface (Supplementary Figure 1; Figure 2C). The viability of all three cell lines decreased in a dose-dependent manner. Colo205 cells were the most sensitive to rhTRAIL, with 10 ng ml^−1^ of rhTRAIL sufficient to decrease cell viability by 61.8±2.3% (Figure 1A). HCT15 and HCA7 cells were less sensitive to rhTRAIL. In these cell lines, a maximal decrease in cell viability to 65.5±3.6% and 80.3±3.0%, respectively, was achieved following treatment with 50 ng ml^−1^ of rhTRAIL. No further decrease in viability was observed with rhTRAIL concentration >50 ng ml^−1^ (Figure 1B and C).

### TRAIL activates the JNK pathway in colon cancer cell lines via both DR4 and DR5

To examine whether the JNK pathway was activated during TRAIL-induced colon cancer cell death, phosphorylation of JNK and its target, c-Jun were assessed by western blot analysis following treatment with rhTRAIL. rhTRAIL (20 ng ml^−1^ for Colo205 and 50 ng ml^−1^ for HCT15 and HCA7 cells) resulted in phosphorylation of JNK in all three cell lines (Figure 2A). Phosphorylation of c-Jun followed a similar pattern (Figure 2B). To elucidate whether JNK phosphorylation was mediated by DR4 or DR5, Colo205 and HCT15 cells were treated with agonistic antibodies against DR4 or DR5. Both cell lines were found to express DR4 and DR5 on the cell surface and ligation of both receptors led to induction of apoptosis (Figure 2C and D). Colo205 cells were more sensitive to the DR5-agonistic antibody (44.3%±3.5 and 27%±1.5 cell death induction by anti-DR5- and anti-DR4 antibodies, respectively). The DR4- and DR5 antibodies (10 nM) induced almost equal levels of apoptosis in HCT15 cells (DR4, 49.8±3.1% and DR5, 45.3±5.7%) measured at 5 h after treatment.

Ligation of both DR4 and DR5 could induce JNK phosphorylation (Figure 2E) in both cell lines. In Colo205 cells, DR5 ligation induced a stronger JNK phosphorylation. This may correlate with the stronger apoptosis-inducing ability of DR5 in these cells (Figure 2E). Agonistic antibody treatment of HCT15 cells demonstrated that the DR4 and DR5 receptors can equally trigger JNK phosphorylation, which was detected maximally after 2 h treatment (Figure 2E). These data suggest that both DR4 and DR5 can induce JNK activation.

### Inhibition of JNK potentiates apoptosis-induction by selective activation of DR4 or DR5, but reduces apoptosis induced by rhTRAIL

**Figure 3:**
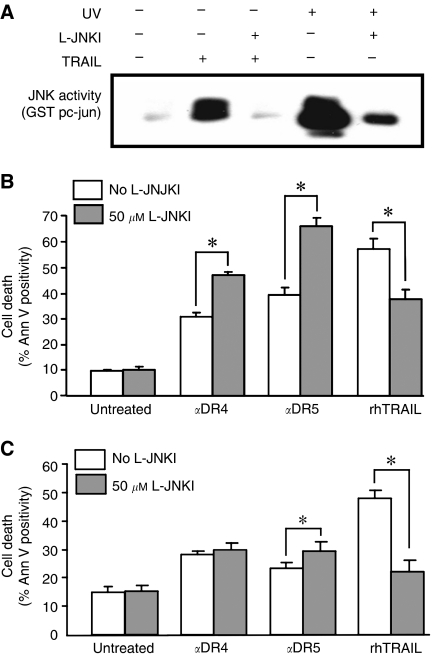
Inhibition of JNK inhibits rhTRAIL-mediated apoptosis, but potentiates DR4- or DR5-mediated apoptosis in colon cancer cells. (**A**) L-JNKI can inhibit rhTRAIL-mediated JNK activation. HCT15 cells were preincubated for 1 h with L-JNKI (25 *μ*M) followed by treatment with 50 ng ml^−1^ rhTRAIL for 3 h or UV for 30 min. The figure shows GST p-c-Jun phosphorylation by JNK as a measure of JNK activity. (**B**) Inhibition of L-JNKI potentiates apoptosis induced by selective activation of DR4 and DR5, but reduces apoptosis induced by rhTRAIL. Colo205 cells were treated for 3 h with either 20 ng ml^−1^ rhTRAIL or 5 nM of DR4/DR5 agonistic antibodies with or without 30 min pretreatment with L-JNKI (50 *μ*M) and induction of apoptosis was measured with Annexin V. The graph shows average cell death±s.e.m. of four independent experiments. The asterisk (^*^) indicates significant differences (*P*<0.05). (**C**) Inhibition of L-JNKI potentiates apoptosis induced by selective activation of DR4 or DR5, but reduces apoptosis induced by rhTRAIL. HCT15 cells were treated for 3 h with either 50 ng ml^−1^ rhTRAIL or 10 nM of DR4/DR5 agonistic antibodies with or without 30 min pretreatment with L-JNKI (50 *μ*M) and induction of apoptosis was measured with Annexin V. The graph shows average cell death±s.e.m. of three independent experiments. The asterisk (^*^) indicates significant differences (*P*<0.05).

In colon cancer cells, the role of JNK in death receptor-induced apoptosis has not been fully elucidated. One of the most selective JNK inhibitors currently available is the JNK-inhibitory peptide analogue, L/D-JNKI (Barr *et al*, 2002) and thus was chosen to inhibit JNK to examine the role of JNK in TRAIL-induced colon carcinoma cell apoptosis. Inhibition of JNK by L-JNKI was confirmed by measuring JNK activity in TRAIL- and UV-treated HCT15 cells with an *in vitro* kinase assay (Figure 3A).

Colo205 and HCT15 cells were treated with rhTRAIL or agonistic DR4/5 antibodies with or without L-JNKI of 50 *μ*M, a concentration generally used due to the short half-life time of the inhibitor pretreatment (Bonny *et al*, 2001). Inhibition of JNK activity in Colo205 cells reduced TRAIL-induced apoptosis (Figure 3B; *P*=0.0041), however enhanced agonistic DR4- (*P*=0.0002) and DR5 antibody-induced cell death (*P*=0.0006; Figure 3B). A similar pattern, albeit to a lesser extent, was observed with HCT15 cells. Inhibition of JNK activity again resulted in a significant increase in DR5 but not DR4 antibody-induced apoptosis (*P*=0.310; *P*=0.041, respectively). On the other hand (as was observed in Colo205 cells), inhibition of JNK reduced rhTRAIL-induced apoptosis (*P*=0.0254; Figure 3C). This surprising result can be explained by either different JNK isoform activation or different JNK compartmentalisation induced by the different treatments.

### Receptor internalisation occurs following treatment with rhTRAIL and agonistic DR4/5 antibodies

**Figure 4:**
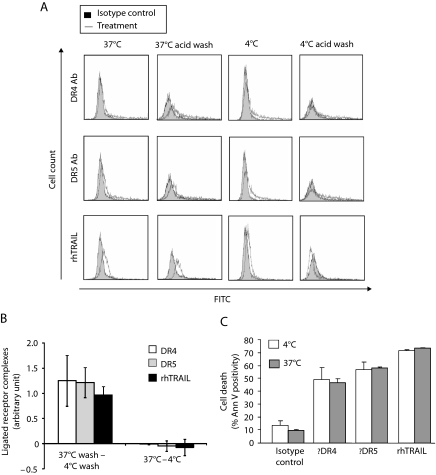
Receptor internalisation induced by rhTRAIL and agonistic DR4/5 antibodies. (**A**) Flow cytometric analysis of ligated receptor internalisation in Colo205 cells. Cells were treated with DR4/5 agonistic antibodies (5 nM) cross-linked with FITC-labelled secondary antibody or FITC-labelled rhTRAIL (20 ng ml^−1^) for 30 min at 37°C or at 4°C. Cells were washed with 0.2 M acetic acid to remove surface bound rhTRAIL and agonistic antibodies. (**B**) Quantification of receptor internalisation. The graph shows the difference of isotype control-normalised MFI measured at 4 and 37°C (normalised MFI at 37°C minus normalised MFI at ^+^4°C). The first set of bars shows the level of receptor internalisation occurred at 37°C after treatment with rhTRAIL or agonistic antibodies (normalised MFI at 37°C/acid wash – normalised MFI at ^+^4°C/acid wash). The second set of bars indicates that rhTRAIL and agonistic DR4- and DR5 antibodies bound to the TRAIL receptors to a similar level regardless of the incubation temperature. (**C**) Induction of apoptosis by treatments detailed in point a. After the 30 min incubation with rhTRAIL or antibodies, the unbound molecules were removed by a wash step and the cells were incubated in normal growth medium for an additional 3 h after which induction cell death was measured with Annexin V. The data shown are representative of three independent experiments.

On ligation by TRAIL, the TRAIL receptor-ligand complexes can be internalised and surface bound *vs* internalised TNF receptors have been shown to induce different signal transduction pathways resulting different cellular responses (Schutze *et al*, 1999; Schneider-Brachert *et al*, 2004; Varfolomeev *et al*, 2005; Kohlhaas *et al*, 2007). We hypothesised that unlike rhTRAIL, agonistic DR4/5 antibodies do not trigger receptor internalisation resulting in JNK activation in a different cellular compartment. Colo205 cells were treated with FITC-labelled rhTRAIL or agonistic DR4/5 antibodies cross-linked by a FITC-labelled secondary antibody for 30 min at either 37°C or ^+^4°C and their internalisation analysed by flow cytometry. An acid wash step was carried out at ^+^4°C after the incubation to remove all non-internalised, surface bound ligand/antibody ensuring that any fluorescent signal was due to internalised ligand/antibody-receptor complexes. The flow cytometric analysis showed that both rhTRAIL and agonistic antibodies bound to the TRAIL receptors at both 37°C and ^+^4°C and were all internalised to a similar extent, when incubated at 37°C but not at ^+^4°C (Figure 4A and B). The same samples were also tested for the ability of rhTRAIL and agonistic antibodies to bind to and activate their receptors in these conditions. At the end of the 30 min incubation, unbound rhTRAIL or agonistic antibodies were removed by a wash step. The samples were incubated for an additional 3 h and induction of apoptosis was detected as a measure of receptor activation. The extent of apoptosis was the same regardless of incubation temperature (Figure 4C), confirming that all treatment conditions enabled ligand/antibody-receptor interaction. These results argue against the compartmentalisation hypothesis.

### rhTRAIL and agonistic DR4/5 antibodies phosphorylate distinct JNK1 isoforms

**Figure 5:**
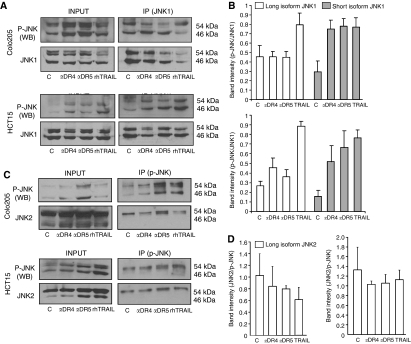
rhTRAIL and selective activation of DR4 or DR5 leads to activation of distinct JNK1 isoforms. (**A**) Phosphorylation of JNK1 isoforms by rhTRAIL and agonistic DR4/5 antibodies. Colo205 cells were treated with 5 nM of agonistic DR4/5 antibodies or 20 ng ml^−1^ rhTRAIL. HCT15 cells treated with 10 nM of agonistic DR4/5 antibodies or 50 ng ml^−1^ rhTRAIL for 3 h. JNK1 was immunoprecipitated (IP) and its phosphorylation pattern was assessed by western blotting with p-JNK antibody. JNK1 western blots show total JNK1 protein immunoprecipitated from cell lysates that was used for quantification of the amount of p-JNKI in the IP. (**B**) Densitometric quantification of p-JNK1 levels. The graph shows averaged p-JNK1 band densities normalised for total JNK1 levels in the immunoprecipitates of Colo205 (top) and HCT15 (bottom) cells from four independent experiments. (**C**) Phosphorylation of JNK2 isoforms by rhTRAIL and agonistic DR4/5 antibodies. Colo205 and HCT15 cells were treated as in point A and p-JNK protein (both JNK1 and 2) was immunoprecipitated. JNK2 phosphorylation was assessed in the p-JNK immunoprecipitates by probing the blots with a JNK2-specific antibody. P-JNK protein levels were also determined for quantification of JNK2 phosphorylation. (**D**) Densitometric quantification of p-JNK2 levels in Colo205 (left) and HCT15 (right) cells. The graph shows averaged p-JNK2 band densities normalised for total JNK2 levels in the immunoprecipitates from two independent experiments.

To address the different effects of JNK inhibition on apoptosis, we next investigated which JNK isoforms were phosphorylated by rhTRAIL and the DR4/5 antibodies. JNK1 was immunoprecipitated from rhTRAIL-treated and agonistic antibody-treated Colo205 and HCT15 cells. Immunoprecipitates were electrophoresed on SDS–PAGE and probed for phosphorylated JNK, to identify which JNK1 isoforms were activated by the different treatments. For quantification, blots were also probed for total JNK1 (Figure 5A) and the densitometric ratio of p-JNK1-long or -short to total JNK1-long or -short was calculated (Figure 5B). In both cell lines, all treatments could initiate phosphorylation of the short JNK1 isoform (46 kDa, JNK1*α*1 and/or JNK1*β*1 isoforms), whereas the long isoform (54 kDa, JNK1*α*2 and/or JNK1*β*2) was only phosphorylated after rhTRAIL treatment (Figure 5A and B). Lysate input from all treated samples confirmed JNK phosphorylation in both cell lines (Figure 5A). Due to the high homology between the *α*1/2 and *β*1/2 isoforms, we could not differentiate between the *α*1/*β*1 and the *α*2/*β*2 isoforms.

To assess which JNK2 isoforms were phosphorylated following selective receptor activation or rhTRAIL treatment, immunoprecipitation with JNK2-specific antibody was attempted, but was unsuccessful due to technical difficulties (no suitable, isoform-specific antibody was available, data not shown). As an alternative strategy, phospho-JNK (including all JNK1 and JNK2 isoforms) was immunoprecipitated, electrophoresed by SDS–PAGE and probed with total JNK2 antibody (this identified whether the short or long JNK2 isoforms were phosphorylated). Total phospho-JNK levels of the same blots were also determined to quantify the levels of phospho-JNK2 isoforms, in a similar manner as for the JNK1 isoforms (Figure 5C and D). The levels of phosphorylated JNK2 did not increase from the baseline after any of the treatments, suggesting that JNK2 is not activated by TRAIL receptors (Figure 5C and D). These data demonstrate that JNK1 is the main JNK subtype activated by TRAIL receptors and selective activation of DR4 or DR5 activated predominantly the short isoforms of JNK1 (JNK1*α*1 and/or JNK1*β*1) whereas rhTRAIL led to phosphorylation of both the short and long JNK1 isoforms (ie JNK1*α*2 and/or JNK1*β*2).

### JNK1*α*1 has an antiapoptotic function

**Figure 6:**
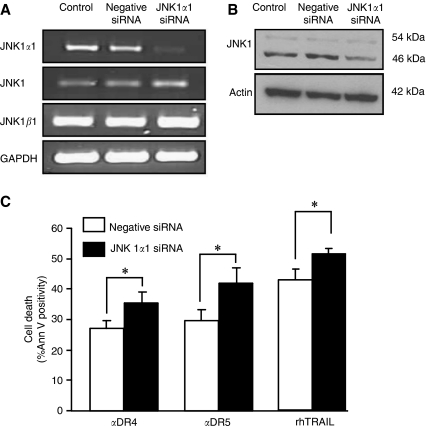
JNK1*α*1 has an antiapoptotic function. (**A**) Knockdown of JNK1*α*1 in Colo205 cells by JNK1*α*1 shRNA expression plasmid. Cells were nucleofected with JNK1*α*1 or scrambled shRNA expression plasmids. Total RNA was isolated 24 h after transfection and JNK expression was detected with primers detecting all JNK1 mRNA isoforms or primers specific to JNK1*α*1 or JNK1*β*1 isoforms. (**B**) Western blot analysis of the expression of the short (JNK1*α*1 and *β*1) and long (JNK1*α*2 and *β*2) isoforms of JNK1 in JNK1*α*1 shRNA expressing cells. (**C**) Knockdown of JNK1*α*1 increases apoptosis induced by selective activation of DR4 or DR5. Colo205 cells were treated with agonistic DR4/5 antibodies (5 nM) or rhTRAIL (20 ng ml^−1^) for 3 h, after which percentage of apoptotic cells was determined with Annexin V. The graph shows the average cell death induced±s.e.m. of five independent experiments (sum of Ann V^+^/PI^−^ and Ann V^+^/PI^+^ percentages). The ^*^ indicates significant differences (*P*<0.05).

The above results indicated that the short isoforms of JNK1 (JNK1*α*1 and/or JNK1*β*1) may play an antiapoptotic role on selective activation of DR4 or DR5. In an effort to dissect its role, Colo205 cells were transiently transfected with an shRNA expression plasmid to JNK1*α*1. RT–PCR demonstrated knockdown of JNK1*α*1 following transient transfection of JNK1*α*1 shRNA, without affecting total JNK1 and JNK1*β*1 mRNA levels confirming isotype-specific knockdown (Figure 6A; isoform specific primer design is depicted in Supplementary Figure 2). Accordingly, a decrease in the protein levels of the short JNK1 isoform was induced by the JNK1*α*1 shRNA observed by western blotting (Figure 6B). Induction of apoptosis by TRAIL or selective induction of DR4 or DR5 by agonistic antibodies was assessed in JNK1*α*1 shRNA transfectants by Annexin V binding. Knockdown of JNK1*α*1 significantly increased apoptosis induced by TRAIL, as well as agonistic DR4- and DR5-antibodies at 3 h of exposure (TRAIL, *P*=0.040; DR4, *P*=0.042; DR5, *P*=0.046) compared to cells transfected with a scrambled shRNA expressing plasmid (Figure 6C). These data suggest that JNK1*α*1 has an antiapoptotic effect.

**Figure 7:**
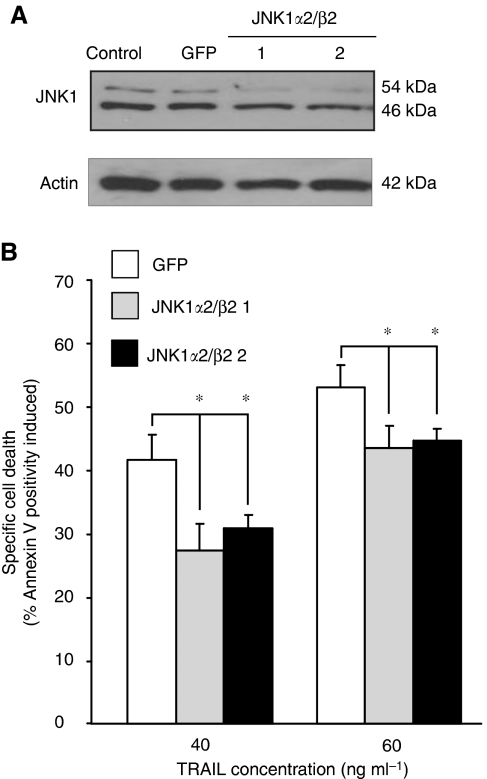
Long JNK1 isoforms have a proapoptotic function. (**A**) Knockdown of JNK1*α*2/*β*2 in Colo205 cells by siRNA. Cells were nucleofected with two different JNK1*α*2/*β*2 siRNAs, or an siRNA against GFP as a negative control. Cell lysates were prepared 24 h after transfection and JNK1-short and -long expression was detected with primers western blotting. (**B**) Knockdown of JNK1*α*2/*β*2 reduces TRAIL-mediated apoptosis. Colo205 cells were treated with rhTRAIL (40 and 60 ng ml^−1^) for 3 h, after which percentage of apoptotic cells was determined with Annexin V. The graph shows the average cell death induced±s.e.m. of three independent experiments (sum of Ann V^+^/PI^−^ and Ann V^+^/PI^+^ percentages). The asterisk (^*^) indicates significant differences (*P*<0.05).

The only region that differs in the long JNK1 isoforms from the short JNK1 isoforms is a 5-nucleotide sequence and thus this was the only region targetable by siRNA. We designed two siRNAs against this region with selectivity for the *α*2/*β*2 isoforms (the targeted region is highlighted in Supplementary Figure 2). The efficiency of the knockdown was analysed by western blotting. Cell lysates of Colo205 cells transfected with JNK1*α*2/*β*2 siRNA or siRNA against GFP as a negative control for 24 h was analysed for JNK1 expression, using a JNK1-specific antibody (Figure 7A). JNK1*α*2/*β*2 siRNAs reduced the expression of the long JNK1 isoforms, without having a non-specific effect on the short JNK1 isoforms. JNK1*α*2/*β*2 siRNA transfected Colo205 cells were then treated with rhTRAIL (40 and 60 ng ml^−1^) for 3 h and induction of apoptosis was measured (Figure 7B). Knockdown of the long JNK1 isoforms reduced TRAIL-induced apoptosis, indicating that these JNK1 isoforms are indeed proapoptotic (40 ng ml^−1^ rhTRAIL, *P*=0.049 and 0.04 for siRNA 1 and 2, respectively; 60 ng ml^−1^ rhTRAIL, *P*=0.047 and 0.04 for siRNA 1 and 2, respectively).

## Discussion

JNK is activated following stimulation of various TNF receptor superfamily members, TNF-R1, Fas, DR4 and DR5 (Sluss *et al*, 1994; Cahill *et al*, 1996; Yang *et al*, 1997; Herr *et al*, 1999). The role of this JNK activation in apoptosis is unclear and opposing, pro- and antiapoptotic functions have been proposed (Bode and Dong, 2007; Yoo *et al*, 2008). Similarly, controversy exists as to the role that activated JNK might play in TRAIL-induced colon carcinoma apoptosis (Zhang *et al*, 2004). This study demonstrates that in colon carcinoma cells that express both DR4 and DR5, both receptors are able to trigger JNK activation and c-Jun phosphorylation. To elucidate the role of JNK activation in DR4- and DR5-mediated apoptosis in colon carcinoma cells, JNK activity was blocked by L-JNKI. L-JNKI was chosen over the widely used SP600125 (Bennett *et al*, 2001) as recent studies found that SP600125 is a rather non-specific JNK inhibitor (Bain *et al*, 2003). Our studies found that inhibition of JNK by L-JNKI reduced rhTRAIL-induced cell death, suggesting a proapoptotic role for JNK. Interestingly, inhibition of JNK potentiated cell death induced by selective activation of DR4 or DR5, suggesting that depending on the type or the total number of receptors activated, a pro- or antiapoptotic JNK signal transduction pathway can be activated.

It has been shown that after binding to its receptors, TRAIL is rapidly internalised by both clathrin-dependent and -independent endocytosis (Kohlhaas *et al*, 2007). Unlike TNF-R1, internalisation is not required for TRAIL-induced apoptosis, as the assembly of the TRAIL DISC already occurs at the cell membrane (Kohlhaas *et al*, 2007). However, internalisation of the ligated receptor has been suggested to play a role in other TRAIL-mediated signalling events such as activation of JNK or NF-*κ*B (Varfolomeev *et al*, 2005). In our hands, internalisation of TRAIL receptors ligated by agonistic DR4 and DR5 antibodies or by rhTRAIL revealed no significant differences; in all cases, the ligated receptor complex was rapidly internalised. This indicates that the opposing apoptosis-modulatory effect of JNK activation induced by TRAIL or selective DR4/DR5 activation was not due to receptor internalisation but possibly different isoforms of JNK activated by the individual receptors.

Ten isoforms of JNK are known to exist as a result of alternative splicing of the three genes, *jnk1*, *jnk2* and *jnk3* (Gupta *et al*, 1996). Little is known about the role of these isoforms in apoptosis. Overexpression of JNK1*β*1 increases resistance to vesicular stomatitis virus-induced cell death in 3T3 fibroblasts, whereas overexpression of JNK1*α*1 and JNK1*β*1 potentiates cisplatin- and doxorubicin-induced cell death in sarcoma cell lines (Han *et al*, 2002; Koyama *et al*, 2006). Previous studies have demonstrated a role for either JNK1 or JNK2 in TNF*α*-induced apoptosis (Dietrich *et al*, 2004; Liu *et al*, 2004). Our results show that the chief JNK isotype activated by DR4 and DR5 is JNK1. Furthermore, whereas TRAIL-mediated receptor activation led to activation of both the long and short isoforms of JNK1, selective ligation of DR4 or DR5 with cross-linked agonistic antibodies predominantly activated the short JNK1 isoforms (JNK1*α*1 and/or *β*1) and the difference in cell death seen following JNK inhibition may be related to the different JNK1 isoforms, with the short isoforms of JNK1 (JNK1*α*1 and JNK1*β*1) transmitting an antiapoptotic signal and the long isoforms of JNK1 (JNK1*α*2 or JNK1*β*2) mediating a proapoptotic signal. As a reason why activation of individual TRAIL receptors by agonistic antibodies activates different JNK isoforms from rhTRAIL, it is likely that the agonistic DR4- and DR5-specific antibodies trigger different receptor clustering, or intracellular conformational changes from those induced by rhTRAIL. It is also possible that on rhTRAIL treatment, higher-order heteromeric receptor complexes (receptosomes) including both DR4 and DR5 are formed where the interaction between the death domains of the various receptor trimers allows for the recruitment of more and/or different adaptor proteins. It has been shown that different agonists of DR4 and DR5 (agonistic antibodies and recombinant ligand) trigger different conformational changes in the receptors resulting in differences in adaptor protein recruitment, such as FADD. This can lead to activation of different downstream signal transduction and kinase pathways (Thomas *et al*, 2004). Our current studies investigate this possibility. Of note, in both Colo205 and HCT15 cells, rhTRAIL was a more efficient inducer of apoptosis than either DR4 or DR5 agonistic antibodies. This may be in line with the hypothesis that full signal transduction requires both DR4 and DR5 activation simultaneously.

To elucidate the roles of the different JNK1 isoforms, JNK1*α*1 expression as well as JNK1*α*2/*β*2 expression was knocked down with shRNA. Knockdown of the short JNK1 isoform potentiated TRAIL-induced apoptosis, whereas elimination of the long JNK1 isoforms blocked rhTRAIL-induced cell death. These results demonstrate that the short JNK1 isoform, JNK1*α*1 acts against apoptosis, whereas the long JNK1 isoforms promote it. In has to be noted, that knocking down JNK efficiently (as JNK is an abundant protein) is very difficult. Even 30–50% of the remaining protein may be sufficient to transmit a signal. Selective targeting one JNK isoform is even more complicated and thus our method probably underestimated the real potency of JNK isoforms in modulating TRAIL-mediated apoptosis.

Genes regulated by JNK1*α*1 and JNK1*β*1 have been previously identified by Han *et al* (2002). Overexpression of a constitutively active form of JNK1*α*1 led to the induction of a number of antiapoptotic/prosurvival genes including proliferin (mitogen-regulated protein), Ack protein tyrosine kinase and serum and glucocorticoid regulated kinase (sgk) and repression of proapoptotic proteins, such as insulin-like growth factor binding protein-4 (IGFBP-4) and suppressor of cytokine signalling-3 (SOCS-3) as a strong indication that JNK1*α*1 indeed triggers antiapoptotic signalling (Han *et al*, 2002). Identifying which of these genes, or other so far unidentified genes, play important role warrants further studies.

In conclusion, we show for the first time that combined activation of all TRAIL receptors *vs* selective activation of DR4 or DR5 result in the activation of different JNK isoforms. Moreover, the short isoform of JNK1, JNK1*α*1, generates an antiapoptotic signal whereas the long isoforms of JNK1 trigger proapoptotic signalling. Our findings shed light on the apparently contradictory results surrounding the role of JNK signalling in TRAIL-induced apoptosis and also suggest a way forward to target cancer cells for sensitisation to killing by inhibition of short isoforms of JNK1.
