# Supplementary figures and images for: Differential activation of JNK1 isoforms by TRAIL receptors modulate apoptosis of colon cancer cell lines
Source: Br J Cancer. 2009 Apr 7;100(9):1415–24. doi: 10.1038/sj.bjc.6605021 (PMC2694422; doi:10.1038/sj.bjc.6605021)

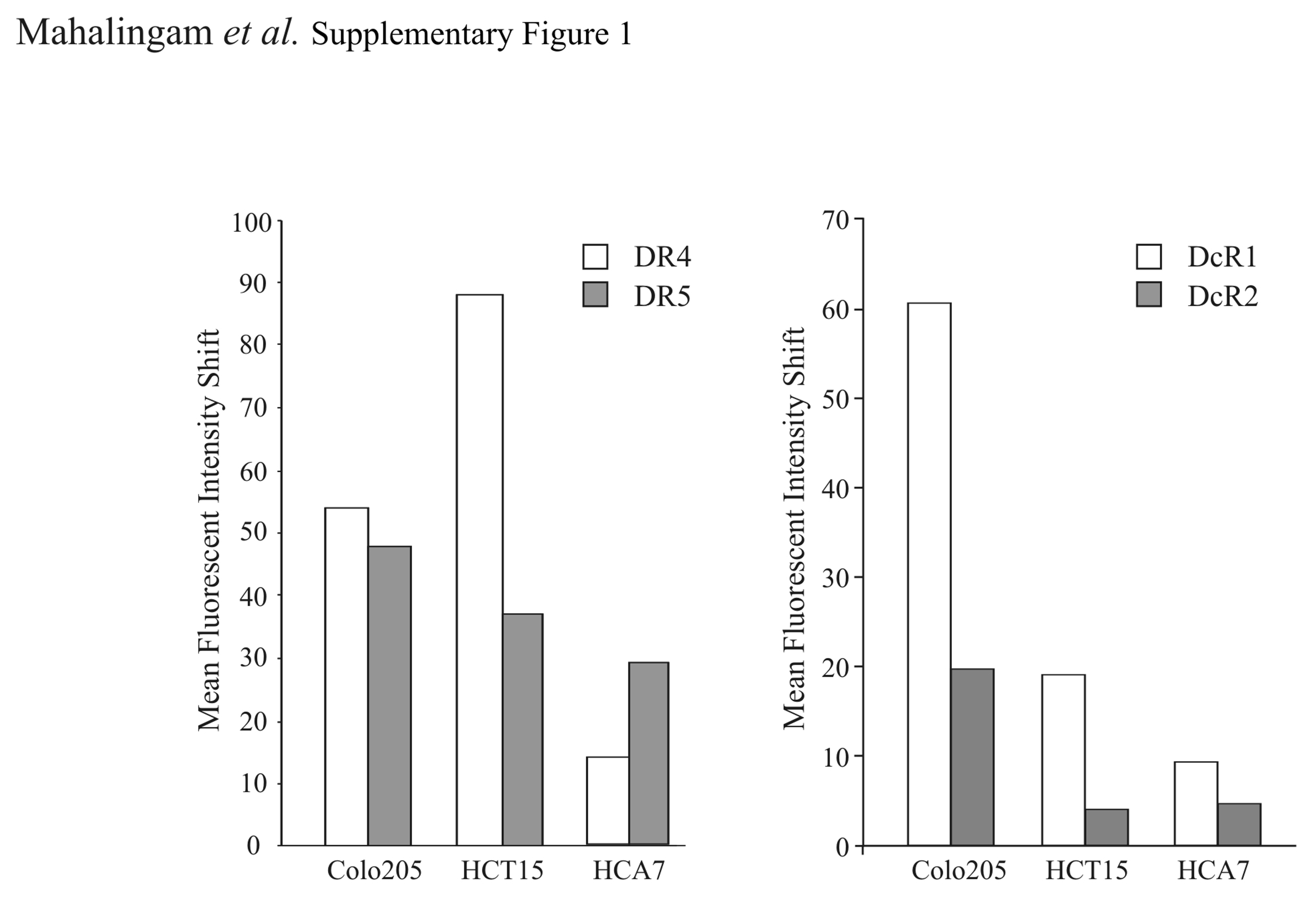

Supplement: Supplementary Figure 1 [file 6605021x1.tif]
