# Supplementary material for: Differential activation of JNK1 isoforms by TRAIL receptors modulate apoptosis of colon cancer cell lines
Source: Br J Cancer. 2009 Apr 7;100(9):1415–24. doi: 10.1038/sj.bjc.6605021 (PMC2694422; doi:10.1038/sj.bjc.6605021)
Supplement: Supplementary Figure 1 Legend [file 6605021x2.doc]

Supplementary Figure 1. DR4, DR5, DcR1 and DcR2 receptor surface expression of Colo205, HCT15 and HCA7 cells. The graph shows mean fluorescence intensity normalized to the isotype control. The graph is a single representation of three independent experiments.
