# Supplementary material for: Differential activation of JNK1 isoforms by TRAIL receptors modulate apoptosis of colon cancer cell lines
Source: Br J Cancer. 2009 Apr 7;100(9):1415–24. doi: 10.1038/sj.bjc.6605021 (PMC2694422; doi:10.1038/sj.bjc.6605021)
Supplement: Supplementary Figure 2 [file 6605021x3.doc]

Mahalingam *et al.* Supplementary Figure 2.

JNK1α1 Forward >>>>>>>>>>>>>>>>>>>>>>>>>>>>

JNK1α1 (NM_002750.2) nt 665 - AGAAATGGTTTGCCACAAAATCCTCTTTCCAGGAAGGGACTATATT

JNK1α2 (NM_139049.1) nt 665 - AGAAATGGTTTGCCACAAAATCCTCTTTCCAGGAAGGGACTATATT

JNK1β1 Forward >>>>>>>>>>>>>>>>>>>>>>> >>>>

JNK1β1 (NM_139046.1) nt 665 - AGAAATGATCAAAGGTGGTGTTTTGTTCCCAGGTACAGATCATATT

JNK1β2 (NM_139047.1) nt 665 - AGAAATGATCAAAGGTGGTGTTTTGTTCCCAGGTACAGATCATATT

JNK1α1 (NM_002750.2) nt 1141 - CCCTCTCCTTTAGCACAGGTGCAGCAGTGATCAATGGCTCTCAGCA

JNK1α2 (NM_139049.1) nt 1141 - CCCTCTCCT**TT** - - - --**AGGTGCAGCAGTGATCA**ATGGCTCTCAGCA

JNK1β1 (NM_139046.1) nt 1141 - CCCTCTCCTTTAGCACAGGTGCAGCAGTGATCAATGGCTCTCAGCA

JNK1β2 (NM_139047.1) nt 1141 - CCCTCTCCT**TT** - - -- -**AGGTGCAGCAGTGATCA**ATGGCTCTCAGCA

<<<<<<<<<<<<<<<<<<<<<<<< JNK1α1/β1 Reverse

Supplementary Figure 2. **Primer design to detect individual JNK1 short isoforms and target sequences of JNK1α2/β2 siRNAs.** Part of the aligned sequence from nucleotide 665-711 and 1141-1186 of JNK1α1, α2, β1 and β2 isoforms are depicted. Blue colour shows nucleotides specific to the β1 and β2 isoforms, while red nucleotides are specific for α1 and β1 isoforms. The binding sites of the different primers are shown above (forward primers) or under the sequences (reverse primers). The target of the JNK1α2/β2 siRNAs is highlighted with bold letters (both siRNAs target the same region, there is only one nucleotide shift between the two target sequences).
